# The Balance Between Intensive and Palliative Care in an Anti-melanoma Differentiation-Associated Gene 5 (MDA5) Antibody-Positive Acute Interstitial Pneumonia in a Nonagenarian: A Case Report

**DOI:** 10.7759/cureus.56983

**Published:** 2024-03-26

**Authors:** Yuri Asano, Taichi Fujimori, Chiaki Sano, Ryuichi Ohta

**Affiliations:** 1 Family Medicine, Shimane University Faculty of Medicine, Izumo, JPN; 2 Community Care, Unnan City Hospital, Unnan, JPN; 3 Community Medicine Management, Shimane University Faculty of Medicine, Izumo, JPN

**Keywords:** anti-mda5 antibody, general physician, rural, myositis-specific autoantibodies, immunosuppressive agents, 80 and over, aged, autoantibodies, interstitial lung diseases, dermatomyositis

## Abstract

This case report details the management of anti-melanoma differentiation-associated gene 5 (MDA5) antibody-positive acute interstitial pneumonia in a 93-year-old man, a condition characterized by rapid progression and high mortality. Despite the grim prognosis typically associated with this disease, especially in elderly patients, the subject of this report survived beyond the expected timeframe, illustrating the effectiveness of prompt and aggressive treatment strategies. Initially presenting with dyspnea, the patient's diagnostic process was challenging due to the absence of dermatomyositis (DM)-specific skin manifestations. However, early suspicion led to the identification of anti-MDA5 antibodies, confirming the diagnosis. The treatment regimen initiated with corticosteroid pulses, cyclophosphamide, tacrolimus, and high-dose gamma globulin therapy significantly improved the patient's respiratory conditions, giving the patient and his family time to decide on their palliative care. This approach underlines the importance of early diagnosis and the implementation of comprehensive treatment strategies in managing anti-MDA5 antibody-positive interstitial pneumonia. In this case, the successful outcome adds valuable insights into the potential for extending survival and enhancing the quality of life in elderly patients with this severe autoimmune condition, emphasizing the need for a proactive and aggressive approach to treatment.

## Introduction

The presence of anti-melanoma differentiation-associated gene 5 (MDA5) antibodies is a significant marker in dermatomyositis (DM), particularly associated with severe progressive interstitial pneumonia [1]. This condition substantially influences patient outcomes and treatment approaches. Understanding the role of these antibodies in the disease's pathogenesis is crucial, yet remains elusive mainly, complicating the development of targeted therapies [2,3]. This case report aims to contribute to the growing literature by detailing the clinical presentation, management, and outcome of a rare anti-MDA5 antibody-positive interstitial pneumonia in a nonagenarian patient.

The prognosis for patients with DM varies significantly based on the presence of specific autoantibodies. Studies have shown that patients with anti-MDA5 antibody-positive DM face a considerably grimmer prognosis compared to those with anti-ARS antibody-positive DM or those negative for both antibodies [4,5]. The stark contrast in survival rates, with a dramatic decline within the first year for anti-MDA5 antibody-positive patients, underscores the aggressive nature of interstitial pneumonia in this group, which often does not respond well to immunosuppressive treatments [6]. This case report explores these prognostic factors further by presenting an even more challenging scenario of an elderly patient, where age-related decline in immune function and increased vulnerability to adverse effects from aggressive treatments compound the difficulty in managing the disease.

This case report of a 93-year-old man with anti-MDA5 antibody-positive interstitial pneumonia represents a unique contribution to the medical literature. It offers insights into the potential for multimodal treatment approaches that avoid the need for invasive procedures like tracheal intubation and give critical patients and their families time for satisfying decision-making. Through this case, we aim to shed light on the complexities of treating acute interstitial pneumonia in the context of anti-MDA5 antibody-positive interstitial pneumonia, especially in elderly patients, and to discuss the balance between intensive and palliative care.

## Case presentation

A 93-year-old male patient sought medical attention at a rural community hospital, presenting with acute dyspnea and dry cough. His dry cough started two weeks ago and gradually exacerbated. Three days ago, he felt dyspnea on exertion. During his initial evaluation on the day of the visit, notable symptoms included cold hands and fingertips, cyanosis, tachypnea, and an oxygen saturation (SpO2) of 81% at a day service facility. He visited his primary care doctor that evening due to persistent symptoms. This visit unveiled a critical drop in his SpO2 levels to 60%-70% and the detection of an abnormal shadow on his left lung, prompting an urgent referral to the rural community hospital. The patient's comprehensive medical history revealed a spectrum of chronic conditions, including hypertension, dyslipidemia, type 2 diabetes mellitus, mild cognitive impairment, facial nerve palsy, and heart failure. His ongoing medications included 5 mg cilnidipine and 100 mg sacubitril valsartan daily.

On the admission day, his vital signs were a Japan Coma Scale (JCSI) of 1, a temperature of 36.7°C, a blood pressure of 156/90 mmHg, a pulse rate of 107 beats/min, a respiratory rate of 22 breaths/min, and an SpO2 of 78% on room air. Physical examination identified bilateral fine crackles across the chest, yet no visible DM-specific cutaneous manifestations such as Gottron's sign or heliotrope rash. Blood gas analysis revealed a partial pressure of carbon dioxide (PCO2) at 32.1 mmHg and a partial pressure of oxygen (PO2) at 53.6 mmHg, confirming type I respiratory failure. The blood tests showed elevated levels of leukocyte count, C-reactive protein (CRP), lactate dehydrogenase (LDH), Krebs von den lungen-6 (KL-6), and surfactant protein A/D (Table 1).

**Table 1:** Initial laboratory data of the patient CRP, C-reactive protein; eGFR, estimated glomerular filtration rate; Ig, immunoglobulin; KL-6, Krebs von den lungen; SP, surfactant protein

Parameter	Level	Reference
White blood cells	11.40	3.5–9.1 × 10^3^/μL
Neutrophils	82.5	44.0–72.0%
Lymphocytes	8.4	18.0–59.0%
Hematocrit	41.5	33.4−44.9％
Mean corpuscular volume	91.0	79.0–100.0 fl
Platelets	40.8	13.0–36.9 × 10^4^/μL
Total protein	8.4	6.5–8.3 g/dL
Albumin	3.3	3.8–5.3 g/dL
Total bilirubin	0.6	0.2–1.2 mg/dL
Aspartate aminotransferase	23	8–38 IU/L
Alanine aminotransferase	20	4–43 IU/L
Lactate dehydrogenase	299	121–245 U/L
Blood urea nitrogen	29.2	8–20 mg/dL
Creatinine	0.93	0.40–1.10 mg/dL
Serum Na	136	135–150 mEq/L
Serum K	4.3	3.5–5.3 mEq/L
Serum Cl	102	98–110 mEq/L
CRP	10.14	<0.30 mg/dL
IgG	1900	870–1700 mg/dL
IgM	61	35–220 mg/dL
IgA	604	110–410 mg/dL
eGFR	57.1	>60.0 ml/min/1.73^2^
KL-6	1938	105.3-401.2 U/ml
SP-A	83.1	<43.8 ng/ml
SP-D	604	<110 ng/ml
Urine test		
Leukocyte	Negative	Negative
Protein	Negative	Negative
Blood	Negative	Negative

**Figure 1:**
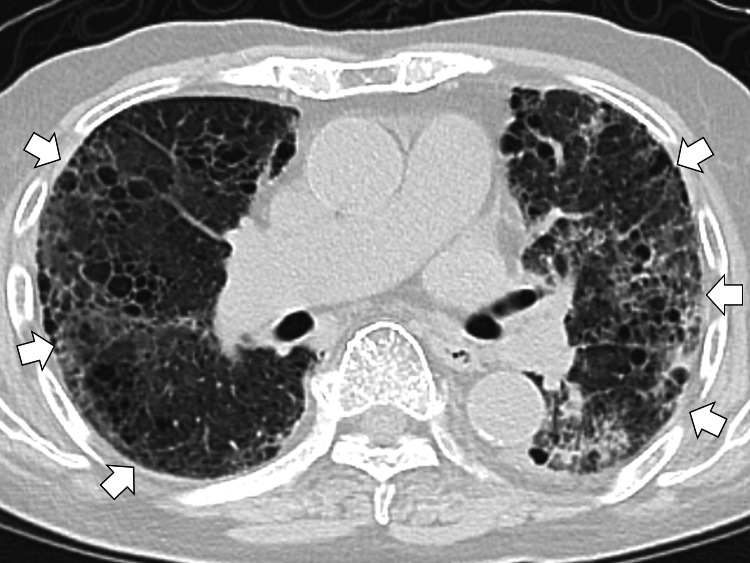
The computed tomography of the chest showing bilateral diffuse interstitial filtration (white arrows)

The chest X-ray showed the left chest infiltrations, showing bacterial pneumonia. The chest computed tomography showed bilateral diffuse interstitial filtration (Figure 1).

The patient's management commenced on day 1 with intravenous ampicillin/sulbactam sodium 3 g/day for suspected bacterial pneumonia. By day 2, due to a lack of significant improvement, the regimen was escalated to include 9 g of ampicillin sodium and 40 mg of water-soluble prednisone intravenously. On the subsequent day, salbutamol was introduced for a possible chronic obstructive pulmonary disease (COPD) exacerbation alongside a substantial dose of intravenous methylprednisolone 1000 mg for an exacerbation of interstitial pneumonia because of serum KL-6 elevation, which demonstrated a marked positive response. This treatment was sustained through days 4 and 5. 

On the sixth day, further diagnostic tests identified elevated levels of anti-MDA5 antibody (index, 48; reference, <32), leading to a diagnosis of anti-MDA5 antibody-positive acute interstitial pneumonia. Serum ferritin was 1021 ng/dL (reference, 39.4-340). In response, an intravenous cyclophosphamide pulse of 500 mg was administered to induce remission, and prophylactic sulfamethoxazole 1 g was initiated to prevent pneumocystis pneumonia. To sustain immunosuppression, oral tacrolimus 3 mg daily and high-dose gamma globulin therapy for five days were commenced on day 7. Following the tailored treatment plan, the patient experienced alleviation of respiratory distress, reducing his oxygen requirements to 2 L/min via nasal cannula. 

His overall functional status did not improve to the pre-admission status, necessitating his transfer to a long-term care ward. Here, in consultation with the patient and his family, a decision was made to pursue palliative care, abstaining from further cyclophosphamide administration. Despite initial improvements, the patient's respiratory condition gradually declined, leading to his death two months later in the presence of his family.

## Discussion

In our encounter with the management of a nonagenarian diagnosed with anti-MDA5 antibody-positive acute interstitial pneumonia, we navigated through the complexities of treating a condition with a notoriously rapid progression and high mortality rate. Literature indicates that individuals with this condition can succumb to the disease within a month of onset, particularly highlighting the aggressive nature of this illness [7,8]. However, our patient's course of disease diverged significantly from this grim prognosis despite his advanced age, which is generally considered a poor prognostic factor for this condition [9]. This divergence underscores the importance of a nuanced approach to diagnosis and treatment in high-risk groups for effective decision-making for patients and their families.

A diagnosis of anti-MDA5 antibody-positive acute interstitial pneumonia presents significant challenges, particularly in the absence of classic DM skin lesions, a common scenario that complicates the clinical picture. Previous research emphasizes the rapid progression and potential fatality of interstitial pneumonia associated with anti-MDA5 antibody-positive DM if treatment is delayed, highlighting the critical need for early suspicion and testing for myositis-specific autoantibodies (MSAs) upon diagnosis of interstitial pneumonia [10,11]. Our case reinforces this approach, demonstrating the value of prompt MSA measurement in patients with interstitial pneumonia, even when traditional DM skin manifestations are absent.

Regarding treatment, the refractory nature of anti-MDA5 antibody-positive acute interstitial pneumonia necessitates an aggressive initial treatment strategy. Early administration of high-dose corticosteroids combined with potent immunosuppressive agents is advocated to mitigate the disease's rapid progression [10,12]. Our management strategy aligned with these recommendations, initiating corticosteroid pulse therapy following an unresponsive treatment to antimicrobial therapy for presumed pneumonia. This early intervention preceded the definitive diagnosis and was pivotal in preventing the progression to more severe disease stages [13].

Upon confirming the anti-MDA5 antibody-positive acute interstitial pneumonia diagnosis, we escalated the treatment to cyclophosphamide, tacrolimus, and high-dose gamma globulin therapy. This therapeutic regimen reflects the current practice, where a combination of corticosteroid pulses, calcineurin inhibitors, and cyclophosphamide has been proven effective [14]. Furthermore, integrating high-dose gamma globulin therapy in our treatment protocol may have played a crucial role in attenuating the disease's severity, aligning with previous reports suggesting its beneficial impact on patient outcomes [14].

The positive outcome in our patient, characterized by avoiding intubation in the first exacerbation and giving time to the patient and his family for satisfying decision-making, beyond the typically short prognosis associated with anti-MDA5 antibody-positive acute interstitial pneumonia, particularly in very elderly patients, illustrates the potential for tailored, transient, aggressive treatment strategies to alter the course of this otherwise devastating disease [13,15]. It underscores the importance of early and accurate diagnosis, the pivotal role of aggressive initial therapy, and the potential benefits of incorporating high-dose gamma globulin therapy in managing high-risk patients, contributing to satisfying palliative care for patients and their families [16,17]. This case adds valuable insight into the growing body of literature on anti-MDA5 antibody-positive interstitial pneumonia, advocating for a proactive, comprehensive approach to treatment that may improve palliative care in this challenging, hyper-older patient population.

## Conclusions

This case study underscores the potential for improving the quality of life in treating anti-MDA5 antibody-positive acute interstitial pneumonia, even among the elderly, through prompt and aggressive therapeutic interventions. The timely application of corticosteroid pulses, followed by a regimen of cyclophosphamide, tacrolimus, and high-dose gamma globulin therapy, demonstrated a pivotal role in avoiding the initial intubation of a 93-year-old patient and giving time for satisfying decision-making of palliative care. These findings highlight the importance of rapid diagnosis and the initiation of an assertive treatment approach, suggesting a promising avenue for enhancing the quality of palliative care in older patients afflicted with this aggressive disease.
